# Investigation of a group of Iranian theater artists' mental health and attitude toward patients with mental disorders

**DOI:** 10.3389/fpubh.2022.990815

**Published:** 2022-09-15

**Authors:** Negin Eissazade, Zahra Aeini, Rozhin Ababaf, Elham Shirazi, Mahsa Boroon, Hesam Mosavari, Adele Askari-Diarjani, Ala Ghobadian, Mohammadreza Shalbafan

**Affiliations:** ^1^Student Research Committee, School of Medicine, Iran University of Medical Sciences, Tehran, Iran; ^2^Department of French Language and Literature, Faculty of Letters and Human Sciences, Shahid Beheshti University, Tehran, Iran; ^3^Mental Health Research Center, Department of Psychiatry, Psychosocial Health Research Institute, School of Medicine, Iran University of Medical Sciences, Tehran, Iran; ^4^Clinical Research Development Unit, 22 Bahman Hospital, Qazvin University of Medical Sciences, Qazvin, Iran

**Keywords:** social stigma, community psychiatry, mental illness, mental health, art

## Abstract

**Introduction:**

Stigmatizing attitude toward patients with severe mental disorders is one of the main obstacles of improving the mental health of societies. Media plays an important role in how the public views mental health issues. Thus, we have performed this study to investigate the Iranian theater artists' mental health status, and their view toward patients with severe mental disorders.

**Methods:**

This cross-sectional study was performed *via* an online anonymous survey including the Social Distance Scale and the Dangerousness Scale measuring the attitude of participants toward patients with severe mental disorders, and the 28-item General Health Questionnaire (GHQ-28). It was disseminated to artists who had the experience of working in theater in the past year in Iran.

**Results:**

Our survey was responded by 104 artists. Social Distance Scale scores' mean was 10.67 (scores can range from 0 to 21) and the Dangerousness Scale scores' mean was 28.87 (scores can range from 8 to 56); higher scores indicate worse discrimination. Our participants' strongest fears were to let someone with a severe mental disorder to take care of their children, and for these groups of patients to obtain a hunting license. Twenty-six (25%) participants were at risk of moderate to severe anxiety, and 18 (17.3%) participants were at risk of moderate to severe depression.

**Conclusion:**

By and large, our participants did not have a positive attitude toward patients with severe mental disorders. Providing the knowledge of mental health issues can help the general public to be more tolerant of the mentally ill and specifically, theater can be employed to fight stigmatizing mental health issues by educating its audience.

## Introduction

Stigma is defined as disapproval of an individual or group based on their distinguishing characteristics. Stigmatizing mental health issues exists on three levels: individual, interpersonal, and institutional. It stems from misconception and prompts falsely applied stereotypes, prejudice, and discrimination (1, 2).

Discrimination takes different forms and may emerge as social disapproval and exclusion. It may lead to decline in social status, worsening the illness, aggression, interpersonal conflicts, and isolation. Furthermore, stigma may encourage substance abuse and reluctance toward treatment. Consequently, people with mental health issues do not just have to carry the burden of their symptoms but also deal with reduced quality of life (1–3).

Previous studies have reported that stigma invigorates the general public to withhold help from minorities (4). Protest, education, and contact are the three suggested components against stigma (5). Some studies have indicated that educating the public with an accurate perception of mental health issues makes stigmatizing less likely (6). Several studies have reported improved attitudes as the outcome of educational programs, which can be used for a wide age range (6–10). There also have been reports of positive results attributed to the public being in contact with patients with severe mental disorders (11).

Media plays a vital role in how the public views mental health issues, and its imprecise representation in television and films has been reinforcing the negative stereotypes. It has been portraying the mentally ill as potentially dangerous people to society (12).

As theater creatively interacts with its audience, it can either be used to fight mental disorders stigma or prompt the negative attitude toward patients with severe mental disorders. Several studies indicated the theater's positive effects on reducing stigma among teenagers and young adults (13–19). There have been reports of the possibility for the role of the dramaturg (13), live presentations (14), applied drama (15), performing arts (16), and theatrical presentations (17) in reducing stigma. A review for evaluating the impact of mass media interventions including film, photographs, radio and comics reported that art interventions are generally effective when they use multiple art forms, but with a small effect (18).

Previous studies have reported that significant rates of moderate to severe mental health issues exist among artists in different fields (19–21) which probably affects their work and what they present to the public. There also have been reports that one's mental health status, especially experiencing depressive symptoms, can affect their attitude toward the mentally ill (22). Considering the significant effect of the theater on public attitude toward patients with severe mental disorders, we conducted this study to evaluate a group of Iranian theater artists' mental health status and attitude toward this group of patients, and to investigate the possible link between these items.

## Methods

### Design

This cross-sectional study was performed *via* an online anonymous survey including the Persian versions of three questionnaires: the Social Distance Scale, the Dangerousness Scale, and the 28-item General Health Questionnaire (GHQ-28).

### Data collection

The survey was open from June of 2021 until June of 2022, and was disseminated to 340 artists through social media (*via* email and chat applications). We used the snowball sampling method, starting at art centers and art schools based in Tehran. Thereafter artists across the country were contacted. The sample size was calculated with CI = 95% (confidence interval), *p* = 0.24 (population proportion) (23) and *d* = 0.09 (sampling error). The inclusion criteria were being over the age of 18, and having the experience of working in theater in the past year.

### Tools

The Social Distance Scale (first developed by Link) and the Dangerousness Scale (first developed by Park) both present cases of patients with severe mental disorders and measure the attitude toward the target person. The Social Distance consists of seven questions and uses the Likert scale as “definitely willing/ probably willing/ probably not willing/ definitely not willing.” The Dangerousness Scale consists of eight questions and uses the Likert scale as “strongly agree, rather agree, agree, nor agree or disagree, disagree, rather disagree, and strongly disagree”. Higher scores indicate worse discrimination (24). Ranjbar Kermani et al. assessed and determined the validity and reliability of the Persian versions of the Social Distance Scale (Cronbach's alpha coefficient: 0.92, test-retest reliability coefficient: 0.89, content validity coefficient: 0.75) and the Dangerousness Scale (Cronbach's alpha coefficient: 0.96, test-retest reliability coefficient: 0.88, content validity coefficients: 0.77) (25, 26).

The 28-item General Health Questionnaire (GHQ-28) includes 28 questions in four subsections measuring the somatic symptoms, anxiety and insomnia, social dysfunction, and depression. Ebrahimi et al. assessed and determined the validity and reliability of the Persian version of the GHQ-28. Its Cronbach's alpha and split reliability co-efficient were 0.78, 0.97 and 0.90 respectively (27).

### Ethical considerations

Participants responded to our survey voluntarily and anonymously. Our study was approved by the Institutional Review Board of Iran University of Medical Sciences (Reference: IR.IUMS.FMD.REC.1400.362).

### Statistical analysis

Data were analyzed by IBM SPSS Statistics (v. 25.0). To report the frequencies and percentages of categorical variables, descriptive statistics were used, and only valid percentages are reported. The demographic data and the GHQ subscales were compared with the variables of the Social Distance Scale and the Dangerousness Scale, through Chi-Square Test.

## Results

A total of 104 artists, within the age range of 18-56, responded to our survey, and more than half (59.6%, *N* = 62) of them were male. Table 1 presents the participants sociodemographic characteristics in detail.

**Table 1:** Sociodemographic characteristics of the participants.

**Gender**
Male	62 (59.6%)
Female	42 (40.4%)
Age	18–56 (Mean: 29.50, Median: 30.00)
**Marital status**
Single/divorced/widow	79 (75.9%)
In a relationship/married	25 (24.0%)
**Role in theater**
Actor	65 (62.5%)
Director	27 (25.9%)
Scriptwriter	15 (14.4%)
Others	4 (3.8%)
**Educational degree**
Masters or higher	81 (77.9%)
Bachelor's degree or lower	23 (22.1%)
**History of visiting a psychiatrist**
Yes	50 (48.0%)
No	54 (51.9%)
History of receiving psychopharmacological treatment
Yes	27 (25.9%)
No	77 (74.0%)
**History of receiving non-psychopharmacological treatment**
Yes	22 (21.1%)
No	82 (78.8%)
**History of admission in a psychiatric ward/hospital**
Yes	1 (0.09%)
No	103 (99.0%)

We have presented the GHQ subscales' score in Table 2. Participants' Social Distance Scale scores ranged from 0 to 21 (Mean: 10.67, Median 10.0, SD: 4.922), and their Dangerousness Scale scores ranged from 11 to 54 (Mean 28.87, Median: 29.00, SD: 10.291). The responses to the Social Distance Scale, and the Dangerousness Scale are presented in Tables 3, 4, respectively.

**Table 2:** Subscales' scores and total scores of the 28-item General Health Questionnaire (GHQ-28).

	**No/very low disorder to mild (*N*, %)**	**Moderate to severe (*N*, %)**
Somatic symptoms	94 (90.3%)	10 (9.6%)
Anxiety and insomnia	78 (75%)	26 (25%)
Social dysfunction	99 (95.1%)	5 (4.8%)
Depression	86 (82.6%)	18 (17.3%)
Total score	89 (85.5%)	15 (14.4%)

**Table 3:** Items and total scores of the Social Distance Scale (SDS).

	**Definitely willing** **(*N*, %)**	**Probably willing (*N*, %)**	**Probably not willing (*N*, %)**	**Definitely not willing (*N*, %)**
How would you feel about renting a room in your home to that person?	19 (18.3%)	35 (33.7%)	30 (28.8%)	20 (19.2%)
What do you think about working as a colleague in the same job as that person?	17 (16.3%)	36 (34.6%)	22 (21.2%)	29 (27.2%)
How do you feel if someone like that person is your neighbor?	24 (23.1%)	54 (51.9%)	16 (15.4%)	10 (9.6%)
What do you think about someone like that person taking care of your children for an hour or two?	22 (21.2%)	16 (15.4%)	22 (21.2%)	44 (42.3%)
What do you think about your children marrying someone like that person?	20 (19.2%)	21 (20.2%)	24 (23.1%)	39 (37.5%)
How do you feel about introducing someone like that person to a young lady who is your friend?	20 (19.2%)	29 (27.9%)	25 (24.0%)	30 (28.8%)
How do you feel about advising someone like that person to a friend for a job?	20 (19.2%)	56 (53.8%)	15 (14.4%)	13 (12.5%)
Total score	Range: 0–21, Mean: 10.67, Median 10.0, SD: 4.922		

**Table 4:** Items and total scores of the Dangerousness Scale.

	**Strongly Agree** **(*N*, %)**	**Rather agree (*N*, %)**	**Agree** **(*N*, %)**	**Nor agree or disagree (*N*, %)**	**Disagree** **(*N*, %)**	**Rather disagree (*N*, %)**	**Strongly disagree (*N*, %)**
If a group of former mental patients lived nearby, I would not allow my children to go to the movie theater alone.	12 (11.5%)	7 (6.7%)	14 (13.5%)	18 (17.3%)	13 (12.5%)	11 (10.6%)	29 (27.9%)
If a former mental patient applied for a teaching position at a grade school and were qualified for the job, I would recommend hiring him/her.	22 (21.2%)	22(21.2%)	20 (19.2%)	16 (15.4%)	8 (7.7%)	16 (15.4%)	0
One important thing about mentally ill people is that you could not say what they will do in the next minute.	16 (15.4%)	8 (7.7%)	17 (16.3%)	22 (21.2%)	11 (10.6%)	14 (13.5%)	16 (15.4%)
If I knew someone had been mentally ill before, I would be less likely to trust them.	6 (5.8%)	12 (11.5%)	16 (15.4%)	18 (17.3%)	18 (17.3%)	13 (12.5%)	21 (20.3%)
The main purpose of psychiatric hospitals is to protect the community from the dangers of the mentally ill people.	13 (12.5%)	2 (1.9%)	11 (10.6%)	9 (8.7%)	11 (10.6%)	17 (16.3%)	41 (39.4%)
If a former mental patient lived nearby, I would not hesitate to allow young children under my care on the sidewalk.	20 (19.2%)	13 (12.5%)	26 (25.0%)	21 (20.2%)	10 (9.6%)	14 (13.5%)	0
Although some mentally ill people may look very good, it is dangerous to forget for a moment that they are mentally ill.	9 (8.7%)	5 (4.8%)	14 (13.5%)	20 (19.2%)	19 (18.3%)	15 (14.4%)	22 (21.2%)
There should be a law forbidding a former mental patient the right to obtain a hunting license.	35 (33.7%)	33 (22.1%)	11 (10.6%)	19 (18.3%)	6 (5.8%)	3 (2.9%)	7 (6.7%)
Total score	Range:11–54, Mean 28.87, Median: 29.00, SD: 10.291		

No significant correlation was found between the demographic data, the GHQ subscales' scores, and the items of the Social Distance Scale, and the Dangerousness Scale.

## Discussion

By and large, our participants' attitude toward patients with severe mental disorders was not positive. The Social Distance Scale scores' mean was 10.67 (±4.922), and the Dangerousness Scale scores' mean was 28.87 (±10.291). We found no significant correlation between the demographic data and the Social Distance Scale scores and the Dangerousness Scale scores, probably due to our small sample size. However, previous studies have reported that older age and marital status (being married) were indicators of negative attitude, and younger age, being female, and higher education were indicators of positive attitude toward patients with severe mental disorders (28–32).

Among the Social Distance Scale items, the question “*What do you think about someone like that person taking care of your children for an hour or two?”* received the most negative feedback. This result was unexpected as there has been no substantial report of child abuse by patients with severe mental disorders over the past years. Notwithstanding that prevention of any type of child abuse or assault is a critical issue among all societies, no rationale supports this fear, and it seems to stem from general attitudes. Besides, previous studies have reported that most child abuse perpetrators are among the families or acquaintances (33).

And among the Dangerousness Scale items, the statement “*There should be a law forbidding a former mental patient the right to obtain a hunting license”* received the most negative feedback. Over the past years in Iran, no murder report with a gun by patients with severe mental disorders has been recorded, which may be because private ownership of guns is illegal. The mass media has propagated the use of guns by these patients over time, especially in the United States of America, and this false image has affected the attitude of different populations and societies. Moreover, previous studies have reported that it is more likely for patients with severe mental disorders to become the victim rather than becoming the offender (34).

As we gathered, 26 (25%) participants were at risk of moderate to severe anxiety, and 18 (17.3%) participants were at risk of moderate to severe depression. In total, 15 (12.6%) participants were at risk of having moderate to severe mental disorders. Whereas, in a study conducted by Noorbala et al. among Iranian general population, using the same cut-offs, it was reported that 29.50% were at risk of anxiety, and 10.39% were at risk of depression. In total, 23.44% of the general population were suspected of moderate to severe mental disorders (23). Low rates of mental disorders among our participants are probably for the reason that people with low levels of anxiety and social dysfunction (a consequence of depression) enter the field of theater, and also, we had a small sample size. Moreover, Kegelaers et al. reported from the Netherlands, that 30% of the electronic music artists (19) and 51.6% of the classical musicians (20) experienced symptoms of depression/anxiety which is much higher than our result (12.6%). In addition, Topoglu et al. reported from Turkey, that 36% of the Turkish state symphony orchestras musicians were at risk of moderate/severe mental health issues, which also holds a higher prevalence than our study (21). All three studies had used the GHQ-12 questionnaire. The difference between our studies was probably due to this fact that the artists working in theater are required to be socially functional to qualify in the field. Also, the GHQ is a screening questionnaire rather than a diagnostic one.

We did not find any significant correlation between our participants' mental health status and their attitude toward patients with psychiatric disorders. However, a study conducted in Finland, reported that dealing with depressive symptoms, leads to a positive attitude toward people with depression (22).

### Strengths and limitations

To the best of our knowledge, this is the first study in Iran to investigate the view of the artists working in theater on mental disorders. Our study is limited by a small sample size, social-desirability bias, and participation bias (participating in a study about psychiatric disorder may have also been a reason for holdback). Probably due to our small sample size, no significant correlation was found between the demographic data, the GHQ subscales' scores, the Social Distance Scale scores, and the Dangerousness Scale scores.

### Implications for practice, research, and policies

Most anti-stigma interventions and campaigns have been conceptualized using knowledge-attitude-behavior paradigm (18), i.e., experiential learning (learning through reflection on doing), empathy building, interactive and prolonged exposure to anti-stigma content (35, 36).

Further investigations should be done among artists, and if needed, we can provide them with anti-stigma activities and interventions, i.e., workshops, screening films or performing plays about mental disorders, and discussion classes that have been suggested by previous studies for other groups (13–18).

## Conclusion

We concluded that all in all, our participants do not have a positive attitude toward patients with severe mental disorders. However, we found no significant correlation between the demographic data, the GHQ-28 scores, the Social Distance Scale scores and the Dangerousness Scale scores, which was probably due to our small sample size. Twenty-five percent of the participants were at risk of moderate to severe anxiety, and 17.3% of the participants were at risk of moderate to severe depression. Our participants' strongest fears were to let patients with severe mental disorders take care of their children, and obtain a hunting license. As reported before, providing knowledge of mental health issues can help the general public to be more tolerant of patients with severe mental disorders. Thus, theater can be employed to fight stigmatizing mental health issues by educating its audience through its creative ways.

## Data availability statement

The raw data supporting the conclusions of this article will be made available by the authors, without undue reservation.

## Ethics statement

The studies involving human participants were reviewed and approved by the Institutional Review Board of Iran University of Medical Sciences (Reference: IR.IUMS.FMD.REC.1400.362). The patients/participants provided their written informed consent to participate in this study.

## Author contributions

Conceptualization and design: NE, ZA, ES, and MS. Data collection: NE, ZA, RA, MB, AA-D, AG, and MS. Data analyses: NE and HM. Initial draft preparation: NE and MS. All authors contributed to the article, editing and review, and approved the submitted version.

## Conflict of interest

The authors declare that the research was conducted in the absence of any commercial or financial relationships that could be construed as a potential conflict of interest.

## Publisher's note

All claims expressed in this article are solely those of the authors and do not necessarily represent those of their affiliated organizations, or those of the publisher, the editors and the reviewers. Any product that may be evaluated in this article, or claim that may be made by its manufacturer, is not guaranteed or endorsed by the publisher.

## References

[1] Adiukwu F Bytyçi DG Hayek SE Gonzalez-Diaz JM Larnaout A Grandinetti P. Global perspective and ways to combat stigma associated with COVID-19. Indian J Psychol Med. (2020) 42:569–74. 10.1177/0253717620964932 33354085 PMC7735248

[2] Rezvanifar F Shariat SV Shalbafan M Salehian R Rasoulian M. Developing an educational package to improve attitude of medical students toward people with mental illness: a delphi expert panel, based on a scoping review. Front Psychiatry. (2022) 13:860117. 10.3389/fpsyt.2022.860117 35360140 PMC8964120

[3] Movahedi S Shariat SV Shalbafan M. Attitude of Iranian medical specialty trainees toward providing health care services to patients with mental disorders. Front Psychiatry. (2022) 13:961538. 10.3389/fpsyt.2022.961538 35966498 PMC9366058

[4] Corrigan PW Rowan D Green A Lundin R River P Uphoff-Wasowski K. Challenging two mental illness stigmas: personal responsibility and dangerousness. Schizophr Bull. (2002) 28:293–309. 10.1093/oxfordjournals.schbul.a006939 12693435

[5] Corrigan PW Penn DL. Lessons from social psychology on discrediting psychiatric stigma. Am Psychol. (1999) 54:765–76. 10.1037/0003-066X.54.9.765 10510666

[6] Link BG Cullen FT. Contact with the mentally ill and perceptions of how dangerous they are. J Health Soc Behav. (1986) 27:289–302. 10.2307/2136945 3559124

[7] Keane M. Contemporary beliefs about mental illness among medical students: implications for education and practice. Acad Psychiatry. (1990) 14:172–7. 10.1007/BF03341291 24430349

[8] Corrigan PW River LP Lundin RK Penn DL Uphoff-Wasowski K Campion J. Three strategies for changing attributions about severe mental illness. Schizophr Bull. (2001) 27:187–95. 10.1093/oxfordjournals.schbul.a006865 11354586

[9] Morrison JK Cocozza JJ Vanderwyst D. An attempt to change the negative, stigmatizing image of mental patients through brief reeducation. Psychol Rep. (1980) 47:334. 10.2466/pr0.1980.47.1.334 7422777

[10] Penn DL Guynan K Daily T Spaulding WD Garbin CP Sullivan M. Dispelling the stigma of schizophrenia: what sort of information is best? Schizophr Bull. (1994) 20:567–78. 10.1093/schbul/20.3.567 7973472

[11] Corrigan PW Edwards AB Green A Diwan SL Penn DL. Prejudice, social distance, and familiarity with mental illness. Schizophr Bull. (2001) 27:219–25. 10.1093/oxfordjournals.schbul.a006868 11354589

[12] Rössler W. The stigma of mental disorders: a millennia-long history of social exclusion and prejudices. EMBO Rep. (2016) 17:1250–3. 10.15252/embr.201643041 27470237 PMC5007563

[13] Rossiter K Gray J Kontos P Keightley M Colantonio A Gilbert J. From page to stage: dramaturgy and the art of interdisciplinary translation. J Health Psychol. (2008) 13:277–86. 10.1177/1359105307086707 18375632

[14] Faigin D Stein C. Comparing the effects of live and video-taped theatrical performance in decreasing stigmatization of people with serious mental illness. JMH. (2008) 17:594–606. 10.1080/09638230701505822

[15] Roberts G Somers J Dawe J Passy R Mays C Carr G. On the edge: a drama-based mental health education programme on early psychosis for schools. Early Interv Psychiatry. (2007) 1:168–76. 10.1111/j.1751-7893.2007.00025.x

[16] Twardzicki M. Challenging stigma around mental illness and promoting social inclusion using the performing arts. J R Soc Promot Health. (2008) 128:68–72. 10.1177/1466424007087804 18402176

[17] Michalak EE Livingston JD Maxwell V Hole R Hawke LD Parikh SV. Using theatre to address mental illness stigma: a knowledge translation study in bipolar disorder. Int J Bipolar Disord. (2014) 2:1. 10.1186/2194-7511-2-1 25505692 PMC4215813

[18] Gaiha SM Salisbury TT Usmani S Koschorke M Raman U Petticrew M. Effectiveness of arts interventions to reduce mental-health-related stigma among youth: a systematic review and meta-analysis. BMC Psychiatry. (2021) 21:364. 10.1186/s12888-021-03350-8 34294067 PMC8296649

[19] Kegelaers J Jessen L Van Audenaerde E Oudejans RR. Performers of the night: Examining the mental health of electronic music artists. Psychol Music. (2022) 50:69–85. 10.1177/0305735620976985

[20] Kegelaers J Schuijer M Oudejans RR. Resilience and mental health issues in classical musicians: a preliminary study. Psychol Music. (2021) 49:1273–84. 10.1177/0305735620927789

[21] Topoglu O Karagulle D. General Health status, music performance anxiety, and coping methods of musicians working in Turkish State symphony orchestras: a cross-sectional study. Med Probl Perform Art. (2018) 33:118–23. 10.21091/mppa.2018.2019 29868686

[22] Esa Aromaa. Attitudes Towards People With Mental Disorders in a General Population in Finland. Helsinki: National Institute for Health and Welfare (THL) (2011). p. 118.

[23] Noorbala AA Faghihzadeh S Kamali K Bagheri Yazdi SA Hajebi A Mousavi MT. Mental Health Survey of the Iranian Adult Population in 2015. Arch Iran Med. (2017) 20:128–34. 28287805

[24] Link BG Cullen FT Frank J woznik JF. The social rejection of former mental patient understanding why labels matter. Am J Psychol. (1987) 92:1461–500. 10.1086/228672

[25] Ranjbar Kermani F Mazinani R Fadaei F Dolatshahi B Rahgozar M. Psychometric properties of the Persian version of social distance and dangerousness scale s to investigate stigma due to severe mental illness in Iran. IJPCP. (2015) 21:254–61.

[26] Rezvanifar F Shariat S V Amini H Rasoulian M Shalbafan M. A scoping review of questionnaires on stigma of mental illness in Persian. IJPCP. (2020) 26:240–56 10.32598/ijpcp.26.2.2619.1

[27] Ebrahimi A Molavi H Moosavi G Bornamanesh A Yaghobi M. Psychometric properties and factor structure of Generl Health Questionnaire 28 (GHQ-28) in Iranian psychiatric patients. J Res Behav Sci. (2007) 5:5–11.

[28] Lin CH Lai TY Chen YJ Lin SK. Social distance towards schizophrenia in health professionals. Asia Pac Psychiatry. (2021) 16:e12506. 10.1111/appy.12506 34915596

[29] Pranckeviciene A Zardeckaite-Matulaitiene K Marksaityte R Endriulaitiene A Tillman DR Hof DD. Social distance in Lithuanian psychology and social work students and professionals. Soc Psychiatry Psychiatr Epidemiol. (2018) 53:849–57. 10.1007/s00127-018-1495-0 29453748

[30] Anosike C Aluh DO Onome OB. Social distance towards mental illness among undergraduate pharmacy students in a Nigerian University. East Asian Arch Psychiatry. (2020) 30:57–62. 10.12809/eaap1924 32611829

[31] Sowislo JF Gonet-Wirz F Borgwardt S Lang UE Huber CG. Perceived dangerousness as related to psychiatric symptoms and psychiatric service use – a vignette based representative population survey. Sci Rep. (2017) 7:45716. 10.1038/srep45716 28367993 PMC5377934

[32] Jalali N Tahan S Moosavi SG Fakhri A. Community attitude toward the mentally ill and its related factors in Kashan, Iran. Int Arch Health Sci. (2018) 5:140–4 10.4103/iahs.iahs_11_18

[33] Youth, Villages,. In Child Sexual Abuse, Strangers Aren't the Greatest Danger, Experts Say. ScienceDaily. Availabel online at: www.sciencedaily.com/releases/2012/04/120413100854.htm (accessed April 13, 2012).

[34] Fazel S Sariaslan A. Victimization in people with severe mental health problems: the need to improve research quality, risk stratification and preventive measures *World Psychiatry* (2021) 20:437–8. 10.1002/wps.20908 34505358 PMC8429327

[35] Walsh DAB Foster JLH. A call to action. a critical review of mental health related anti-stigma campaigns. Front Public Health. (2021) 8:1–15. 10.3389/fpubh.2020.569539 33490010 PMC7820374

[36] Mellor C. School-based interventions targeting stigma of mental illness: systematic review. Psychiatr Bull. (2014) 38:164–71. 10.1192/pb.bp.112.041723 25237538 PMC4115419

